# Differentiation between Glioblastoma Multiforme and Primary Cerebral Lymphoma: Additional Benefits of Quantitative Diffusion-Weighted MR Imaging

**DOI:** 10.1371/journal.pone.0162565

**Published:** 2016-09-15

**Authors:** Ching Chung Ko, Ming Hong Tai, Chien Feng Li, Tai Yuan Chen, Jeon Hor Chen, Ginger Shu, Yu Ting Kuo, Yu Chang Lee

**Affiliations:** 1 Department of Medical Imaging, Chi Mei Medical Center, Tainan, Taiwan; 2 Institute of Biomedical Science, National Sun Yat-Sen University, Kaohsiung, Taiwan; 3 Department of Pathology, Chi Mei Medical Center, Tainan, Taiwan; 4 Graduate Institute of Medical Sciences, Chang Jung Christian University, Tainan, Taiwan; 5 Department of Radiology, E-DA Hospital, I-Shou University, Kaohsiung, Taiwan; George Washington University, UNITED STATES

## Abstract

The differentiation between glioblastoma multiforme (GBM) and primary cerebral lymphoma (PCL) is important because the treatments are substantially different. The purpose of this article is to describe the MR imaging characteristics of GBM and PCL with emphasis on the quantitative ADC analysis in the tumor necrosis, the most strongly-enhanced tumor area, and the peritumoral edema. This retrospective cohort study collected 104 GBM (WHO grade IV) patients and 22 immune-competent PCL (diffuse large B cell lymphoma) patients. All these patients had pretreatment brain MR DWI and ADC imaging. Analysis of conventional MR imaging and quantitative ADC measurement including the tumor necrosis (ADCn), the most strongly-enhanced tumor area (ADCt), and the peritumoral edema (ADCe) were done. ROC analysis with optimal cut-off values and area-under-the ROC curve (AUC) was performed. For conventional MR imaging, there are statistical differences in tumor size, tumor location, tumor margin, and the presence of tumor necrosis between GBM and PCL. Quantitative ADC analysis shows that GBM tended to have significantly (P<0.05) higher ADC in the most strongly-enhanced area (ADCt) and lower ADC in the peritumoral edema (ADCe) as compared with PCL. Excellent AUC (0.94) with optimal sensitivity of 90% and specificity of 86% for differentiating between GBM and PCL was obtained by combination of ADC in the tumor necrosis (ADCn), the most strongly-enhanced tumor area (ADCt), and the peritumoral edema (ADCe). Besides, there are positive ADC gradients in the peritumoral edema in a subset of GBMs but not in the PCLs. Quantitative ADC analysis in these three areas can thus be implemented to improve diagnostic accuracy for these two brain tumor types. The histological correlation of the ADC difference deserves further investigation.

## Introduction

The differentiation between glioblastoma multiforme (GBM) and primary cerebral lymphoma (PCL) is clinically important because treatment strategies of these two different diseases are substantially different. In patients with GBM, wide surgical resection followed by irradiation therapy and chemotherapy with temozolomide is the treatment of choice, whereas patients with PCL usually need to receive high-dose methotrexate-based chemotherapy with or without irradiation after stereotactic biopsy [1–3]. Conventional magnetic resonance imaging (MRI) allows differentiation between these two diseases because the appearance of GBM is usually a ring-shaped contrast-enhanced mass lesion with central hypointense necrosis on contrast-enhanced T1-weighted MR imaging (T1WI), whereas PCL usually present as a solid mass lesion with homogeneous contrast enhancement in immunocompetent patients [4, 5]. However, this pattern is not reliable in some clinical scenarios because atypical, solid enhancing GBM without visible necrosis may mimic PCL, and atypical PCL with visible necrosis may mimic GBM [3].

Although some studies have shown statistically significant differences in apparent diffusion coefficient (ADC) values on diffusion-weighted MR imaging (DWI) between the GBM and PCL [3, 6–9], others have reported that ADC might not be helpful due to substantial overlapping or the absence of statistically significant difference in values for these two diseases [10–15]. Furthermore, it has been observed that the ADC values in peritumoral edema play an important role in the differentiation of brain tumors [16–20]. To the best of our knowledge, no literature has yet described the differences in ADC between GBM and PCL in components including tumor necrosis, the most strongly-enhanced tumor area, and peritumoral edema on diffusion-weighted MR imaging. The purpose of this article is to describe the MR imaging characteristics of GBM and PCL with emphasis on the quantitative ADC analysis in the tumor necrosis, the most strongly-enhanced tumor area, and the peritumoral edema.

## Materials and Methods

### Ethics statement

The retrospective study was approved by the Institutional Review Board of the Chi Mei Medical Center (approval reference number: 10503–010). Oral consent for use of clinical records was taken from patients during follow-up. Written consent was not obtained since the retrospective study did not affect the healthcare of the included individuals. All patients’ records/information were anonymized and de-identified prior to analysis.

### Patient selection

The inclusion criteria in this study are patients with pretreatment brain MR DWI and ADC imaging and were sequentially diagnosed with either primary GBM or PCL in pathology. From July 2006 to June 2015, 121 patients were diagnosed with GBM (WHO grade IV) confirmed by biopsy in two hospitals (Chi Mei Medical Center and E-Da Hospital), and 10 patients were excluded because of pretreatment MR imaging were not available. 7 patients were excluded because DWI or ADC images were not available. Finally, 104 patients (58 men, 46 women, aged 22–87 years; median age, 60 years) were included. For the selection of appropriate patients with PCL, only previously untreated, immunocompetent patients were included. 28 patients were diagnosed with PCL (diffuse large B cell lymphoma). Only 26 patients had pretreatment MR images, and 4 patients were excluded because DWI or ADC images were not available. In total, 22 patients with PCL (9 men, 13 women, aged 38–77 years; median age, 59 years) met the aforementioned inclusion criteria.

### MR Imaging

The MRI images in this study were acquired using a 1.5T (Siemens, MAGNETOM Avanto)(n = 56; 46 for GBM and 10 for PCL) or a 1.5T (GE Healthcare, Signa HDxt) MR scanner (n = 70; 58 for GBM and 12 for PCL) equipped with a 12-channel head coil. The protocols of 1.5T (Siemens, MAGNETOM Avanto) MR imaging were as the following: axial T1-weighted spin-echo (T1WI)(TR/TE, 2000/7 ms; FOV, 16–22 cm; slice thickness/spacing, 5 mm/6.5 mm; matrix, 320 x 228), T2-weighted imaging (T2WI) (fast spin-echo)(3730/108 ms; FOV, 16–22 cm; slice thickness/spacing, 5 mm/6.5 mm; matrix, 384 x 261), fluid attenuated inversion recovery (FLAIR) (9000/92 ms; FOV, 16–22 cm; slice thickness/spacing, 5 mm/6.5 mm; matrix, 256 x 209), and T2-weighted gradient-recalled echo (GRE) (830/26 ms; FOV, 16–22 cm; slice thickness/spacing, 5 mm/6.5 mm; matrix, 256 x 168). Contrast-enhanced images obtained in axial and coronal T1WI (1950/8 ms; FOV, 16–23 cm; slice thickness/spacing, 5 mm/6.5 mm; matrix, 320 x 216) were performed after intravenous administration of 0.1 mmol/kg of body weight of gadobutrol (Gadovist; Schering, Berlin, Germany) or gadoterate meglumine (Dotarem; Guerbet, Villepinte, France). The DWI was performed by applying sequentially in the *x*, *y*, and *z* directions with the following parameters: TR/TE, 3800/105 ms, flip angle, 90 degrees, slice thickness/spacing, 5 mm/6.5 mm; *b* = 0, 500, and 1000 sec/mm^2^. ADC maps were obtained from these imaging data.

The protocols of 1.5T (GE Healthcare, Signa HDxt) MR imaging were as following: axial T1WI (TR/TE, 2000/11 ms; FOV, 20–20 cm; slice thickness/spacing, 5 mm/6.5 mm; matrix, 320 x 192), T2WI (3600/108 ms; FOV, 20–20 cm; slice thickness/spacing, 5 mm/6.5 mm; matrix, 320 x 224), FLAIR (9002/140 ms; FOV, 20–20 cm; slice thickness/spacing, 5 mm/6.5 mm; matrix, 256 x 160), and T2-weighted GRE (450/15 ms; FOV, 18–24 cm; slice thickness/spacing, 5 mm/6.5 mm; matrix, 288 x 160). Contrast-enhanced axial and coronal T1WI (2131/8 ms; FOV, 18–20 cm; slice thickness/spacing, 5 mm/6.5 mm; matrix, 288 x 160) after intravenous administration of 0.1 mmol/kg of body weight of Gadovist or Dotarem. The DWI was performed by applying sequentially in the *x*, *y*, and *z* directions with the following parameters: TR/TE, 6600/73 ms; flip angle, 90 degrees, slice thickness/spacing, 5 mm/6.5 mm; *b* = 0 and 1000 sec/mm^2^.

### Image Analysis

#### Conventional MR imaging features

Two experienced neuroradiologists (T.Y.C., a neuroradiologist with 15 years of experience, and Y.C.L., a neuroradiologist with 14 years of experience) performed qualitative and quantitative imaging measurement of GBMs and PCLs on conventional and diffusion-weighted MR analysis. In cases of multifocal tumors, the lesion largest in size was chosen for measurement. In judging tumor location for large tumors involving more than one location, the dominant location with the largest tumor volume was determined by consensus.

#### Visual Inspection of DWI signals

Qualitative visual inspection of DWI rating of GBM and PCL was performed by T.Y.C. and Y.C.L. by consensus. Each lesion was evaluated as being predominantly hyperintense, isointense, or hypointense relative to the cerebral white matter, and the findings were recorded.

#### ADC Measurement

The necrotic component of the tumor was identified on contrast-enhanced axial T1WI as the interior hypointense, non-enhancing part of the mass lesion. Peritumoral edema was identified as the peritumoral hyperintense area on axial T2 FLAIR MR images. The most strongly-enhancing area of the tumor was identified as mass lesions with the strongest enhancement on contrast-enhanced axial T1WI. The regions of interest (ROI) were placed in a way to avoid volume averaging with cystic and hemorrhagic regions that might influence the ADC values. Cystic components were differentiated from necroses as round-shaped regions with smooth borders exhibiting uniform signal as the cerebrospinal fluid. Hemorrhagic lesions were identified as hyperintense in T1WI or the presence of susceptibility artifacts on T2-weighted GRE. Circular ROIs with areas ranging from 30 to 76 mm^2^ were placed within the necrotic area of the tumor (ADCn), the most strongly-enhanced area of the tumor (ADCt), and the peritumoral edema (ADCe) for obtaining measurements in all cases of GBMs and PCLs. The largest lesion was measured for patients with multifocal tumors. For the peritumoral edema, the ROI was placed in the middle of the largest edematous area for measurement. Because the peritumoral edema varies in size and each edematous location may show varying ADC values, further ADC gradients in the peritumoral edema were measured in a subset of GBMs and PCLs. For a subset of GBMs or PCLs with edematous area larger than 3cm in length and 2cm in width, three separate ROIs with the same area (40 mm^2^) were placed consecutively in the peritumoral edema from the most proximal location near the enhancing tumor to the peripheral edematous region adjacent to normal-appearing white matter. The ROI in the middle of the peritumoral edema (halfway between the margin of enhancing tumor to the border of peritumoral edema) was called e2. The ROI in the middle between the margin of enhancing tumor and e2 was called e1 (a quarter of the length from the margin of enhancing tumor to the border of peritumoral edema), and the ROI in the middle between e2 and the border of peritumoral edema was called e3 (three- quarters of the length from the margin of enhancing tumor to the border of peritumoral edema). We recorded the ADC values from these ROIs (ADCe1, ADCe2, and ADCe3) and calculated the ADC gradients in the peritumoral edema as the subtractions ADCe2—ADCe1, ADCe3—ADCe2, ADCe3—ADCe1. Each area was measured three times by two neuroradiologists (T.Y.C. and Y.C.L.), and the values were averaged for subsequent analysis.

### Statistical Analysis

Statistical analyses were performed using statistical package SPSS for Windows (V.20.0, IBM, Chicago, Illinois, USA). Inter-observer reliability for categorical data (conventional MR imaging features and DWI signals) was determined by using the Cohen kappa (k) coefficient, whereas for continuous data (tumor size and ADC measurement), the intraclass correlation coefficient (ICC) was calculated with the two-way random model and absolute agreement on average measures. Interpretation of the Cohen k and ICC were interpreted according to methods described by Landis and Koch [21] as follows: less than 0, poor; 0.0–0.20, slight; 0.21–0.40, fair; 0.41–0.60, moderate; 0.61–0.80, substantial; and 0.81–1.00, almost perfect. For evaluating the imaging manifestations of GBM and PCL on conventional MR and DWI, Fisher exact test was performed for categorical data and Wilcoxon–Mann–Whitney test was performed for continuous data. Receiver operating characteristic (ROC) analysis with optimal cut-off values was performed for each parameter (ADCn, ADCt, and ADCe) and the combination of parameters to reach sensitivity, specificity and accuracy for discriminating between GBM and PCL. Area-under-the ROC curve (AUC) values for discrimination were also calculated. P-values < 0.05 were considered statistically significant.

## Results

### Conventional MR imaging features

The conventional MR imaging features of GBMs and PCLs are summarized in Table 1. The median tumor size of GBM is larger than that of PCL. Although both tumors occur most commonly in the frontal lobe, there is a statistically significant difference in the location of these two diseases. Daughter nodules, which are small nodules surrounding the main tumor lesion with or without connection, occurred in 22 (21.2%) GBM patients but in only 1 (4.5%) PCL patient. Tumor necrosis is commonly seen in GBMs (97.1%); by contrast, it is seen in only 8 PCL patients (36.4%). All of the GBMs and PCLs show contrast enhancement of the main tumor mass. There is statistically significant difference (P<0.05) between GBMs and PCLs when it comes to tumor size, tumor location, tumor margin, and presence of tumor necrosis. For inter-observer reliability, Cohen k values between 0.75 to 1 for categorical MR imaging and ICC of 0.96 (95% confidence interval: 0.93, 0.98) for tumor size indicate substantial to almost perfect reproducibility.

**Table 1:** The Demographic Data and Conventional MR Imaging Features of GBMs and PCLs.

	GBM (n = 104)	PCL (n = 22)	P-value
**Age (y/o), median (Q1,Q3)**	60 (47, 70.3)	59 (54.3, 71.8)	0.57
**Gender, n (%)**			0.24
Male	58 (55.8%)	9 (41%)	
Female	46 (44.2%)	13 (59%)	
**Metastasis at diagnosis**			0.32
Yes	1 (1%)	1 (4.5%)	
No	103 (99%)	21 (95.5%)	
**Tumor size (cm), median (Q1,Q3)**	5.2 (4, 6)	3.5 (2.6, 4.3)	< 0.01*
**Tumor location, n (%)**			0.02*
Frontal	38 (36.5%)	9 (41%)	
Parietal	25 (24%)	3 (14%)	
Temporal	27 (26%)	2 (9%)	
Occipital	3 (2.9%)	0 (0)	
Basal ganglia	2 (1.9%)	4 (18%)	
Corpus callosum	3 (2.9%)	2 (9%)	
Thalamus	5 (4.8%)	1 (4.5%)	
Brainstem	1 (1%)	1 (4.5%)	
**Tumor margin, n (%)**			**<** 0.01*
Smooth	5 (4.8%)	10 (45%)	
Irregular	99 (95.2%)	12 (55%)	
**Multiplicity, n (%)**			0.26
Yes	21 (20.2%)	7 (32%)	
No	83 (79.8%)	15 (68%)	
**Daughter nodule, n (%)**			0.08
Yes	22 (21.2%)	1 (4.5%)	
No	82 (78.9%)	21 (95.5%)	
**Tumor necrosis, n (%)**			**<** 0.01*
Yes	101 (97.1%)	8 (36.4%)	
No	3 (2.9%)	14 (63.6%)	
**Tumor enhancement, n (%)**			1
Yes	104 (100%)	22 (100%)	
No	0	0	
**Peritumoral edema, n (%)**			1
Yes	97 (93.3%)	21 (95.5%)	
No	7 (6.7%)	1 (4.5%)	

* Statistical difference (P<0.05) in Fisher exact test or Wilcoxon–Mann–Whitney test.

### DWI signals

**Fig 1:**
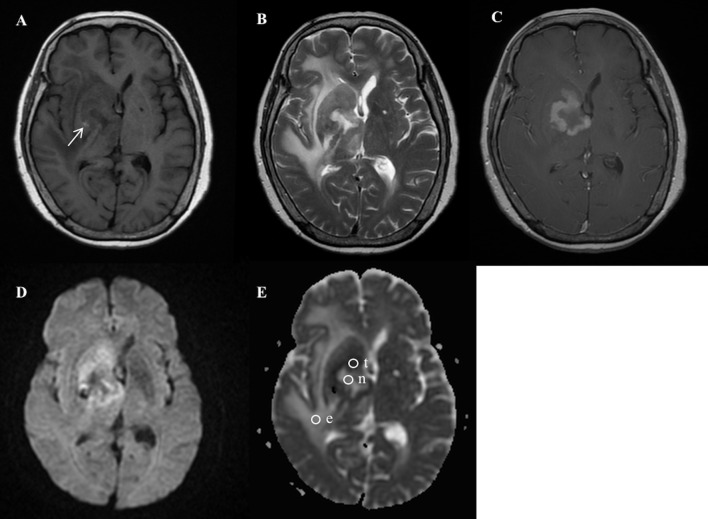
A 74-year-old female with pathologically-proven primary cerebral lymphoma after biopsy. (A) Axial T1-weighted MR imaging (T1WI) and (B) T2-weighted MR imaging (T2WI) showed a heterogeneous tumor mass with central tumor necrosis and peritumoral edema involving the right basal ganglia, right internal capsule, and right thalamus. Spot hemorrhage in the tumor mass is shown as hyperintensity (arrow) on T1WI (A). (C) Axial contrast-enhanced T1WI revealed rim-enhancing mass in the right basal ganglia and internal capsule. (D) Diffusion-weighted imaging (DWI) showed a ring-shaped hyperintense tumor mass, central hypointense necrosis, and isointense peritumoral edema. (E) Apparent diffusion coefficient (ADC) map showed ROIs (circles) in the tumor necrosis (n), the most strongly-enhanced tumor area (t), and the peritumoral edema (e). Measured ADC (b = 1000 sec/mm^2^) in the tumor necrosis (ADCn) is 2.44 x 10^−3^ mm^2^/s; the most strongly-enhanced tumor area (ADCt), 0.84 x 10^−3^ mm^2^/s; the peritumoral edema (ADCe), 2.52 x 10^−3^ mm^2^/s.

**Fig 2:**
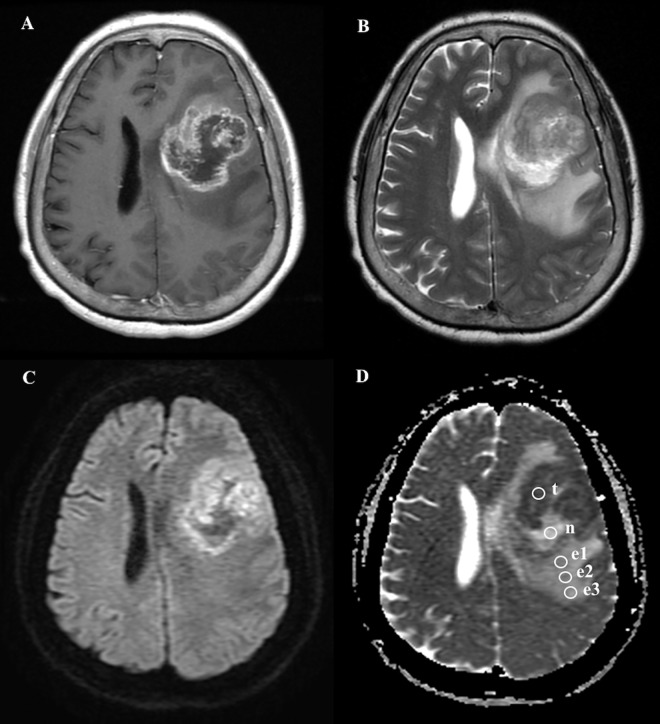
A 64-year-old female with pathologically-proven glioblastoma multiforme after biopsy. (A) Axial contrast-enhanced T1WI showed a tumor mass with rim enhancement and central hypointense non-enhancing necrosis in the left frontal lobe. (B) Axial T2WI showed extensive peritumoral edema associating with the mass lesion. (C) DWI showed a ring-shaped hyperintense tumor mass, central hypointense tumor necrosis, and isointense peritumoral edema. (D) ADC map showed ROIs (circles) in the tumor necrosis (n), the most strongly-enhanced tumor area (t), and the gradient of peritumoral edema from the most proximally-located area (e1) of the peritumoral edema to the most peripheral location (e3). Measured ADC (b = 1000 sec/mm^2^) in the tumor necrosis (ADCn) is 1.85 x 10^−3^ mm^2^/s; the most strongly-enhanced tumor area (ADCt), 0.87 x 10^−3^ mm^2^/s; proximal peritumoral edema (ADCe1), 1.3 x 10^−3^ mm^2^/s; middle peritumoral edema (ADCe2), 1.36 x 10^−3^ mm^2^/s; distal peritumoral edema (ADCe3), 1.43 x 10^−3^ mm^2^/s.

Signals of DWI in the tumor necrosis, the most strongly-enhanced tumor area, and the peritumoral edema of GBM and PCL are listed in Table 2. Most GBMs and PCLs show hypointense DWI signal in the tumor necrosis and hyperintensity in the most strongly-enhanced tumor area as compared to white matter (Figs 1 and 2). As for peritumoral edema, most GBMs (75/97, 77.3%) show isointense DWI signals, but most PCLs (12/21, 57.1%) show hypointense DWI when compared with white matter. There is a statistically significant difference (P<0.05) in the DWI signal of peritumoral edema between these two different tumors. Perfect inter-observer reliability was determined with Cohen’s k value of 0.96.

**Table 2:** Signals of DWI in the Tumor necrosis, The most strongly-enhanced tumor area, and the Peritumoral edema between GBMs and PCLs.

Location / DWI signal	GBM (n = 104)	PCL (n = 22)	P-value
**Tumor necrosis, n (%)**	n = 101	n = 8	0.46
Hypointensity	86 (85.1%)	7 (87.5%)	
Isointensity	4 (4%)	1 (12.5%)	
Hyperintensity	11 (10.9%)	0 (0)	
**The most strongly-enhanced tumor area, n (%)**	n = 104	n = 22	0.5
Hypointensity	3 (2.9%)	0 (0)	
Isointensity	8 (7.7%)	0 (0)	
Hyperintensity	93 (89.4%)	22 (100%)	
**Peritumoral edema, n (%)**	n = 97	n = 21	< 0.01*
Hypointensity	18 (18.6%)	12 (57.1%)	
Isointensity	75 (77.3%)	8 (38.1%)	
Hyperintensity	4 (4.1%)	1 (4.8%)	

* Statistical difference (P<0.05) in Fisher exact test.

### Quantitative ADC values

**Fig 3:**
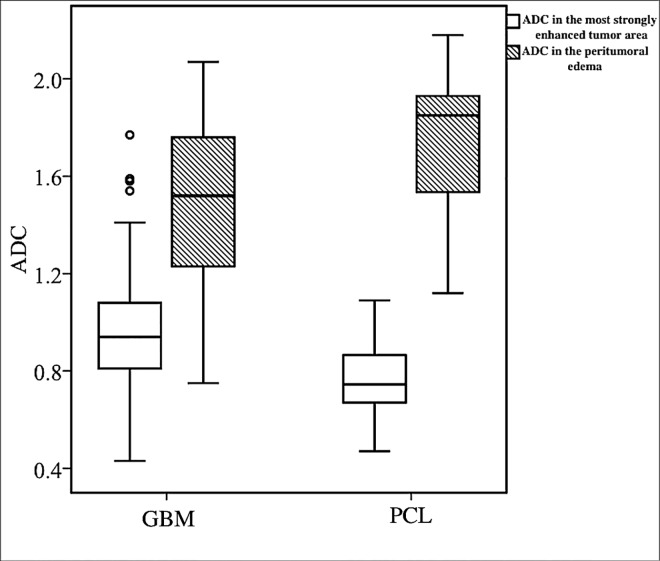
Box plot of diffusion characteristics in patients with GBM and PCL. There is statistically significant difference (P<0.05) between the ADC values (x10^-3^ mm^2^/s, at b = 1000 sec/mm^2^) of GBMs and PCLs in the most strongly-enhanced tumor area (white box) and the peritumoral edema (gray box). Boxes indicate interquartile range, and whiskers indicate range. Circles represent outliers, defined as distances greater than 1.5 times interquartile range below first quartile or above third quartile. In each box, horizontal line represents median.

The ADC values (x10^-3^ mm^2^/s, b = 1000 sec/mm^2^) of GBMs and PCLs in the tumor necrosis (ADCn), the most strongly-enhanced tumor area (ADCt), and the peritumoral edema (ADCe) are summarized in Table 3. The median ADC values of GBMs were higher than PCLs in the most strongly-enhanced tumor area, but lower than PCLs in the peritumoral edema (P<0.05) (Fig 3). The inter-observer reliability showed substantial to almost perfect reproducibility for ADC measurement with ICC of 0.94 (95% confidence interval: 0.88, 0.98) for ADCn, 0.96 (95% confidence interval: 0.91, 0.98) for ADCt, and 0.88 (95% confidence interval: 0.79, 0.95) for ADCe.

**Table 3:** The Median and Quartiles (Q1, Q3) of ADC values (x10^-3^ mm^2^/s, b = 1000 sec/mm^2^) in the Tumor necrosis, The most strongly-enhanced tumor area, and the Peritumoral edema between GBMs and PCLs.

Location	GBM Number / ADC median (Q1, Q3)	PCL Number / ADC median (Q1, Q3)	P-value
**Tumor necrosis (ADCn)**	101 / 1.96 (1.51, 2.51)	8 / 1.69 (1.6, 1.79)	0.25
**The most strongly-enhanced tumor area (ADCt)**	104 / 0.94 (0.81, 1.08)	22 / 0.71 (0.67, 0.86)	< 0.01*
**Peritumoral edema (ADCe)**	97 / 1.52 (1.23, 1.76)	21 / 1.85 (1.55, 1.9)	< 0.01*

* Statistical difference (P<0.05) in Wilcoxon-Mann Whitney test.

### ROC curve analysis

**Fig 4:**
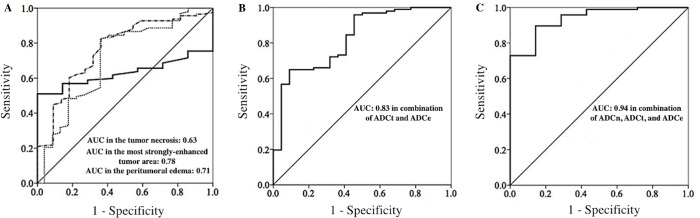
Receiver operating characteristic (ROC) curves of ADC values for differentiation between GBM and PCL. (A) The left diagram represents the ROC curves of ADC values (x10^-3^ mm^2^/s, b = 1000 sec/mm^2^) in the tumor necrosis (ADCn) (full line), the most strongly-enhanced tumor area (ADCt) (dash—dot line), and the peritumoral edema (ADCe) (dot line). The optimal cutoff values of ADCn, ADCt, and ADCe are 1.85, 0.77, and 1.81 respectively. Area under ROC curve (AUC) in the ADCn, ADCt, and the ADCe are 0.63, 0.78, and 0.71 respectively. (B) The middle diagram represents the ROC curve of combined ADCt and ADCe, with AUC value of 0.83. (C) The right diagram represents the ROC curve of combined ADCn, ADCt, and ADCe, with AUC value of 0.94.

The optimal cutoff values of ADC for differentiation between GBM and PCL in the tumor necrosis (ADCn), the most strongly-enhanced tumor area (ADCt), and the peritumoral edema (ADCe) are summarized in Table 4. There is a statistically significant difference (P<0.05) for the area under the ROC curve (AUC) in ADCt and ADCe. Further, the AUC of combined ADCt and ADCe is 0.83 (P<0.05), and the AUC of combined all three parameters is 0.94 (P<0.05) (Fig 4).

**Table 4:** Sensitivity and Specificity in Differentiation of GBMs from PCLs with Receiver Operating Characteristic (ROC) Curve Analysis.

ADC/ Location	Sensitivity	Specificity	AUC	Cut-off value	P-value
ADCn	0.57	1	0.63	1.85x10^-3^mm^2^/s	0.25
ADCt	0.84	0.64	0.78	0.77x10^-3^mm^2^/s	< 0.01*
ADCe	0.83	0.64	0.71	1.81x10^-3^mm^2^/s	< 0.01*
ADCt and ADCe	0.65	0.91	0.83	-	< 0.01*
ADCn, ADCt, and ADCe	0.9	0.86	0.94	-	< 0.01*

* Statistical difference (P<0.05) in differentiation between GBM and PCL.

### ADC gradients in peritumoral edema

**Fig 5:**
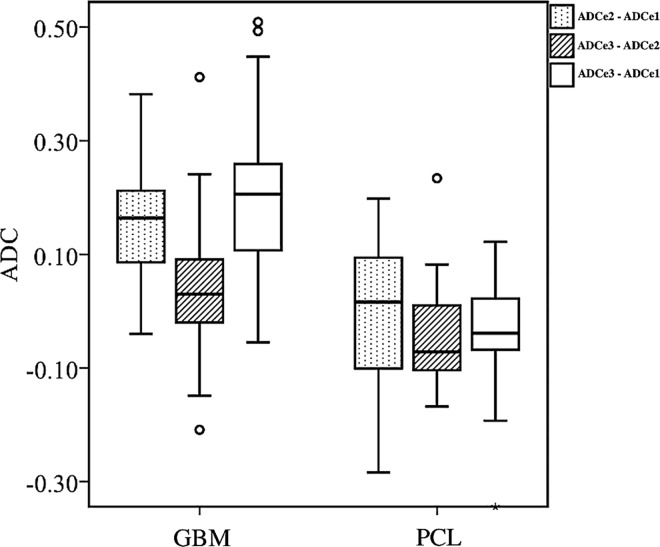
Box plot of the ADC gradients in the peritumoral edema between GBM and PCL.

The ADC gradients in the peritumoral edema in a subset of GBM and PCL (Fig 2) are calculated in Table 5. The ADC values at e1 (the ROI adjacent to the enhancing tumor) are lower in GBM as compared with PCL (P<0.05). Besides, there is also statistical difference (P<0.05) in the gradients of ADCe2—ADCe1, ADCe3—ADCe2, and ADCe3—ADCe1 between GBM and PCL (Fig 5).

**Table 5:** The Median and Quartiles (Q1, Q3) of ADC Gradients in Peritumoral edema in a subset of GBMs and PCLs.

ADC gradients of peritumoral edema	GBM (n = 33) median (Q1, Q3)	PCL (n = 17) median (Q1, Q3)	P-value
ADCe1	1.55 (1.44, 1.65)	1.71 (1.68, 1.84)	< 0.01*
ADCe2	1.72 (1.51, 1.84)	1.82 (1.55, 1.88)	0.39
ADCe3	1.75 (1.59, 1.82)	1.75 (1.54, 1.8)	0.72
ADCe2—ADCe1	0.16 (0.09, 0.21)	0.02 (-0.1, 0.09)	< 0.01*
ADCe3—ADCe2	0.03 (-0.02, 0.09)	-0.07 (-0.1, 0.01)	0.01*
ADCe3—ADCe1	0.21 (0.11, 0.26)	-0.04 (-0.07, 0.02)	< 0.01*

* Statistical difference (P<0.05) in Wilcoxon-Mann Whitney test.

There is a statistically significant difference (P<0.05) in the ADC gradient values (x10^-3^ mm^2^/s, b = 1000 sec/mm^2^) of ADCe2—ADCe1, ADCe3—ADCe2, and ADCe3—ADCe1 between GBM and PCL. Boxes indicate interquartile range, and whiskers indicate range. The outliers are excluded. Circles represent outliers, defined as distances greater than 1.5 times interquartile range below first quartile or above third quartile. In each box, the horizontal line represents the median.

## Discussion

The results from our study have concluded that there are statistically significant differences between GBMs and PCLs when it comes to tumor size, tumor location, tumor margin, and the presence of tumor necrosis on conventional MR imaging. Nonetheless, it is still difficult to differentiate between these two tumors with conventional MR imaging alone in clinical practice, particularly in cases of atypical GBM with smooth tumor margin (5/104, 4.8%) and absence of necrosis (3/104, 2.9%) or atypical PCL with irregular tumor margin (12/22, 55%) and the presence of tumor necrosis (8/22, 36.4%). According to our observations, most GBMs show isointense DWI signal in the peritumoral edema as compared to cerebral whiter matter, but most PCLs show hypointense changes instead (P<0.05). The quantitative ADC analysis show that the median ADC values of GBM are significantly (P<0.0.5) higher than PCL in the most strongly-enhanced tumor area and lower than PCL in the peritumoral edema with cuff-off values of 0.77 x 10^−3^ mm^2^/s and 1.81 x 10^−3^ mm^2^/s, respectively. Although the AUC of ADC values in the most strongly-enhanced tumor area (ADCt) and peritumoral edema (ADCe) for the differentiation between GBM and PCL are only 0.78 and 0.71 (P<0.05) respectively, good AUC (0.83) is found after combining ADCt and ADCe. Furthermore, excellent AUC (0.94) with optimal sensitivity of 90% and specificity of 86% in differentiation between GBM and PCL was obtained by combination of ADCn, ADCt, and ADCe. Finally, there are positive ADC gradients in peritumoral edema in a subset of GBMs but not in the PCLs. Quantitative analysis of ADC values on diffusion-weighted MR imaging thus offers additional values in differentiating these two tumors.

For conventional MR imaging, tumor necrosis is the most often described as a discriminator for the differentiation between GBM and PCL, however, it is difficult to achieve a good differentiation by conventional MR imaging alone [3–5]. The sensitivity of tumor necrosis in predicting GBM in our study is 97.1%, but the specificity is only 63.6%. Analysis of ADC in the tumor necrosis (ADCn) of GBM and PCL has not been reported before, and 0.63 of AUC in ADCn with sensitivity of 57% and specificity of 100% is found in this study. Although there is no statistical significance (P = 0.25) in the AUC of ADCn in differentiation between GBM and PCL, the worth of ADCn arises from combination of ADCn with ADCt and ADCe, which then results in excellent AUC (0.94) with 90% in sensitivity and 86% in specificity for differentiating these two tumors.

On conventional MR imaging, we cannot distinguish these two tumors based on the presence of tumor enhancement because all of GBMs and PCLs showed tumor enhancement in our study. For the most strongly-enhanced tumor area, median ADC value (x 10^−3^ mm^2^/s) of 0.94 in GBM and 0.71 in PCL with cuff-off value of 0.77 are found in our study. Toh et al. [9] reported mean ADC value (x 10^−3^ mm^2^/s) of 0.963 in ten GBM patients and 0.63 in ten PCL patients with cuff-off value of 0.818. Kickingereder et al. [3] reported mean ADC value (x 10^−3^ mm^2^/s) of 0.97 in twenty-eight atypical non-necrotic GBM patients and 0.65 in nineteen PCL patients. Based on these literature reviews, we acknowledge that our study included the greatest number of patients, and the results are in concordance with previous studies [3, 7–9, 22, 23]. Although there is no measurement of contralateral corresponding unaffected white matter for normalization of individual variance in our study, Toh et al. [9] and Horger et al. [24] reported lower ADC values and ADC ratios in tumor mass of PCL as compared with GMB with or without normalization. The lower ADC values in the enhancing tumor of PCL as compared with GBM were due to higher cellularity with relative reduction of the extracellular space for the water molecules to move around (more restricted diffusion) [6, 22].

Peritumoral edema is the T2 high-signal, non-enhancing area that surrounds the enhancing tumor core. It is caused by local breakdown of the blood–brain barrier in the brain tumor, leading to an increased capillary permeability that results in the retention of plasma fluid and protein in the extracellular spaces [25]. Conventional MR imaging shows peritumoral edema in 93.3% of GBM patients and 95.5% of PCL patients in our study, and it is impossible to differentiate these two tumors based only on the presence of peritumoral edema due to poor specificity. The comparison of ADC values in the peritumoral edema between GBM and PCL had only been reported by Server et al. [11] with thirty-seven GBM and five PCL patients, and there was no statistically significant difference in the study. Our study reveals statistically lower ADC values of peritumoral edema in GBM as compared with PCL. Furthermore, there is a statistically significant difference in ADCe1 (proximal peritumoral edema near the enhancing tumor) but not in ADCe2 or ADCe3. According to previous studies, GBM consists of a central tumor mass surrounded by peritumoral invasive malignant cells that decrease in number toward the periphery [16, 26–29]. For PCL, Koeller et al. [30] reported diffuse microscopic tumor infiltrating in peritumoral edema of primary CNS lymphoma in a radiologic-pathologic study. Therefore, peritumoral edema is better referred to as “infiltrative edema” for both GBM and PCL because it represents not only vasogenic edema but also infiltrating tumor cells. The lower ADC values of peritumoral edema (ADCe and ADCe1) in GBM than PCL in our study may be caused by lesser water diffusivity due to more infiltrating tumor cells [25, 31]. Unfortunately, the tumor mass of GBM was removed piece by piece by the neurosurgeon during the operation, and intraoperative biopsy was done only in the tumor mass area for PCL in our study. No intact peritumoral edema tissue was obtained for pathologically confirming the presence of infiltrative tumor cells, and further radiologic-histological correlation is necessary.

In this study, the ADC values are higher in the peritumoral edema than the enhancing tumor for both GBM and PCL. This could be explained by the fact that increased vasogenic edema resulting from the disruption of the BBB in the peritumoral edema causes increased extracellular water and more water diffusivity [17, 25]. For GBM, Lee et al. [17] and Server et al. [11] reported higher ADC values in the peritumoral edema as compared with the enhancing tumor, which are similar to our results. In contrast, Lemercier et al. [16] report lower ADC values in the peritumoral edema than the enhancing tumor in GBMs. For PCL, the comparison of ADC values between the peritumoral edema and the enhancing tumor has not been reported before.

We found positive ADC gradients in peritumoral edema with increased ADC values from near the enhancing tumor (ADCe1) toward the normal cerebral white matter (ADCe3) in a subset of GBM but not in PCL. The positive ADC gradients in peritumoral edema for GBM from our study is consistent with a previous study reported by Lemercier et al. [16], indicating a greater number of malignant cells next to the enhancing tumor than in locations farther from the core lesion [16, 17, 26–28, 32]. For peritumoral edema of PCL, the lower ADCe1 compared with ADCe2 could be caused by more infiltrating tumor cells with less water diffusivity like in GBM. In contrast to GBM, the highest ADC values are in the middle of peritumoral edema (ADCe2) rather than far from the enhancing tumor (ADCe3) for PCL. The decreased ADC value from ADCe2 to ADCe3 in PCL could be explained by decreased water diffusivity due to decreased infiltrative edema toward the normal white matter, but further histological correlation still need to be done. Because this is the first study describing the ADC gradients of peritumoral edema in PCL, no other data is available for correlation.

To the best of our knowledge, this is the first study describing the quantitative ADC in the tumor necrosis, the most strongly-enhanced tumor area, and the peritumoral edema with gradient analysis simultaneously between GBM and PCL on diffusion-weighted MR imaging. This is also the study including the greatest number of cases of GBM and PCL for comparison. Of the various advanced MR imaging, we evaluated the usefulness of only DWI to differentiate GBM from PCL because DWI should be available in most hospitals and is the fast technique without the use of contrast medium. The images are easy to process. However, this study is not without its inherent limitations. As in other ROI-based studies, subjective placement of the ROIs might have influenced the accuracy of the ADC measurements. In addition, neither comparison of histological cellularity in the tumor mass of GBM and PCL nor biopsy of peritumoral edema was performed; therefore, we cannot definitively state that the ADC differences between GBM and PCL are due to differences in cellularity and size of extracellular space. Furthermore, this is a retrospective study including MR imaging performed in earlier period, advanced MR parameters such as perfusion-weighted imaging (DSC-PWI) or susceptibility-weighted imaging (SWI) are not available in some cases to be used for comparison.

## Conclusions

The results from this study demonstrated that there are statistically significant differences between GBM and PCL when it comes to tumor size, tumor location, tumor margin, and presence of tumor necrosis on conventional MR imaging. Quantitative ADC analysis on diffusion-weighted MR imaging shows that GBM tended to have significantly (P<0.05) higher ADC in the strongly-enhanced tumor area and lower ADC in the peritumoral edema area as compared with PCL. Excellent AUC (0.94) with optimal sensitivity of 90% and specificity of 86% for differentiating between GBM and PCL is obtained after the combination of ADC in the tumor necrosis, the most strongly-enhanced tumor area, and peritumoral edema. In addition, there are positive ADC gradients in the peritumoral edema in a subset of GBM but not in PCL. Quantitative ADC values measured in these three areas can thus be implemented to improve diagnostic accuracy for these two brain tumor types. The histological correlation of the ADC difference between these two tumors deserves further investigation in the future.

## Supporting Information

S1 File The Supporting Data of GBM and PCL in this study. (ZIP)
